# Mutations in *LAMB2* causing a severe form of synaptic congenital myasthenic syndrome

**DOI:** 10.1136/jmg.2008.063693

**Published:** 2009-02-20

**Authors:** R A Maselli, J J Ng, J A Anderson, O Cagney, J Arredondo, C Williams, H B Wessel, H Abdel-Hamid, R L Wollmann

**Affiliations:** 1 Department of Neurology, University of California Davis, Davis, California, USA; 2 Department of Neurology, School of Veterinarian Medicine, University of California Davis, Davis, California, 95618, USA; 3 Department of Pediatrics, Neurology Division, Children’s Hospital of Pittsburgh, Pittsburgh, Pennsylvania, USA; 4 Department of Pathology, The University of Chicago, Chicago, Illinois, USA

## Abstract

**Background:**

We describe a severe form of congenital myasthenic syndrome (CMS) associated with congenital nephrosis and ocular malformations caused by two truncating mutations in the gene encoding the laminin β2 subunit (*LAMB2*).

**Methods and results:**

Mutational analysis in the affected patient, who has a history of a serious untoward reaction to treatment with acetylcholinesterase inhibition, revealed two frame-shifting heteroallelic mutations, a maternally inherited *1478delG* and a paternally inherited *4804delC*. An anconeus muscle biopsy demonstrated a profound distortion of the architecture and function of the neuromuscular junction, which was strikingly similar to that seen in mice lacking laminin β2 subunit. The findings included: pronounced reduction of the axon terminal size with encasement of the nerve endings by Schwann cells, severe widening of the primary synaptic cleft and invasion of the synaptic space by the processes of Schwann cells, and moderate simplification of postsynaptic folds and intact expression of the endplate acetylcholinesterase. The endplate potential quantal content was notably reduced, while the frequencies and amplitudes of miniature endplate potentials were only moderately diminished and the decay phases of miniature endplate potentials were normal. Western blot analysis of muscle and kidney tissue and immunohistochemistry of kidney tissue showed no laminin β2 expression.

**Conclusion:**

This case, which represents a new type of synaptic CMS, exemplifies the wide variability of phenotypes associated with *LAMB2* mutations and underscores the fundamental role that laminin β2 plays in the development of the human neuromuscular junction.

Congenital myasthenic syndromes (CMS) represent a diverse group of neuromuscular disorders characterised by varying degrees of muscle weakness and fatigability resulting from impaired neuromuscular transmission. These syndromes are classified into presynaptic, synaptic basal-lamina-associated, and postsynaptic subgroups depending on which compartment of the neuromuscular junction (NMJ) is primarily targeted by the disease.^1^ ^2^ To date, mutations in 11 human genes have been implicated in the pathogenesis of CMS. Mutations causing postsynaptic CMS have been identified in genes encoding both the fetal and the four adult subunits of the acetylcholine receptor (*CHRNG* (MIM 100730), *CHRNA1* (100690), *CHRNB1* (100710), *CHRND* (100720), and *CHRNE* (100725)), rapsyn (*RAPSN* (601592)), MuSK (*MUSK* (601296)), Dok-7 (*DOK7* (610285)), and the skeletal muscle sodium channel (*SCN4A* (603967)).^1^^–^7 In addition, defects in genes encoding choline acetyltransferase (*CHAT* (118490)), and the collagenic tail subunit of the acetylcholinesterase (*COLQ* (603033)) have been found to associate with presynaptic CMS and synaptic basal-lamina-associated CMS, respectively.^8^ ^9^

Mutations in the gene encoding the laminin β2 subunit (*LAMB2* (150325)) have been found to be responsible for Pierson syndrome (MIM 609049), which is characterised by congenital nephrosis and ocular defects.^10^ The majority of the reported *LAMB2* defects are truncation mutations, which result in a complete lack of laminin β2 immunoreactivity in the glomerular basal membrane as well as a severe phenotype that leads to death within days or months after birth.^10^

Laminins are multidomain heterotrimeric glycoproteins of the basal lamina composed of one α, one β, and one γ chains.^11^ In mammals, there are currently five α, four β and three γ isoforms described, which assemble into no fewer than 15 different heterotrimers.^12^ The different laminin isoforms are expressed in a tissue and developmental stage specific manner.^13^ The laminin trimers that are expressed at the NMJ are primarily laminin-4, -9 and -11, which all contain the β2 chain encoded by *LAMB2*.^14^ The essential role that laminin β2 plays in the organisation of the NMJ is demonstrated by the observation that mice lacking laminin β2 *(Lamb2^−/−^)* show defective neuromuscular synapses and die within the first weeks of life.^15^ In these laminin β2^−/−^ mutants, Schwann cell processes, which normally cap the NMJ without entering into the synaptic space, intrude into the primary cleft and impair neuromuscular transmission.^15^

## PATIENT AND FINDINGS

Our patient, who is currently a 20-year-old woman, was born to non-consanguineous parents. During the neonatal period she experienced several episodes of respiratory distress, and she was found to have persistently constricted pupils and massive proteinuria. A renal biopsy at that time demonstrated findings consistent with a microcystic nephrosis. All of her motor developmental milestones were delayed, and although her nephrotic syndrome was corrected at the age of 15 months after a successful kidney transplant from her father, her motor deficit persisted. At the age of 7 years a muscle biopsy revealed non-specific changes, but an electromyogram with repetitive stimulation at 3 Hz of the left median nerve resulted in a 24% decrement of the compound muscle action potential amplitude, which became more pronounced with higher frequencies of stimulation. Serum antibodies against the acetylcholine receptor (AChR) were negative, and a brain magnetic resonance image (MRI) was normal. A trial with a cholinesterase inhibitor resulted in profound weakness requiring hospitalisation and ventilatory support; however, she responded well to treatment with ephedrine. When she was 9 years old she required corrective surgery for severe ptosis. At that time an ophthalmologic examination revealed pronounced miosis, myopia and impaired visual acuity in both eyes. Fundoscopic examination revealed hypoplastic macular areas and poor foveal reflex, but her electroretinogram was normal. A neurologic examination revealed normal cognition, impaired visual acuity and reactive pinpoint pupils. Her external ocular movements were limited to 15–20° in the horizontal axis, and she had bilateral ptosis. She showed no bulbar deficit, but the motor examination revealed severe proximal limb weakness. She had spine surgery for scoliosis at the age of 12, and since then she has used bi-level positive airway pressure during night sleep and intermittently during the day.

To elucidate the nature of the CMS in this patient, we performed an anconeus muscle biopsy at age 10, which included in vitro microelectrode recordings as previously described.^16^ The most remarkable finding of the microelectrode recordings was the profound reduction of the quantal content of end plate potentials (EPPs) evoked by nerve stimulation at 1 Hz relative to the controls (table 1, t = −5.88, p<0.001). Nerve stimulation at higher frequencies resulted in an even more severe reduction of the EPP quantal content as shown by the estimate of the ratio of EPP quantal content using 20 Hz stimulation to 1 Hz stimulation, which was decreased relative to controls. Frequencies and amplitudes of miniature endplate potentials (MEPPs) were also diminished (t = 3.55, p<0.05). However, the half-decay phases of MEPPs were not different from recordings performed in age matched controls (table 1). At two endplates MEPPs were recorded under voltage clamp conditions. The decay time constants of the resultant miniature endplate currents (MEPCs) were not different from controls (mean (SEM) 3.71 (0.15) ms, n = 2 in the patient; and 3.58 (0.16) ms, n = 11 in controls). Finally, to verify that the endplate acetylcholinesterase was active we measured the amplitudes and half-decay phases of the MEPPs recorded before and after exposure of the preparation to 0.1 μM neostigmine. The average amplitude and half-decay phase of MEPPs recorded before neostigmine exposure, 1.16 (0.67) mV and 2.94 (0.67) ms, respectively, increased to 1.42 (0.62) mV and 6.08 (0.88) ms after exposure of the muscle to neostigmine, thus indicating that the endplate acetylcholinesterase was active.

**Table 1:** Physiological data

	Patient	Control
MEPPs amplitude (mV)	1.04 (0.12)* (*n* = 10)	1.58 (0.13) (*n* = 18)
MEPP decay (ms)	3.81 (0.33) (*n* = 10)	3.14 (0.19) (*n* = 18)
MEPP frequency (MEPPS/min)	1.59 (0.30)* (*n* = 9)	5.55 (1.07) (*n* = 11)
EPP quantal content (1 Hz)	2.21 (0.43)** (*n* = 5)	12.61 (1.20) (*n* = 18)
EPP quantal content (20 Hz/1 Hz)	0.65 (0.067)* (*n* = 5)	1.00 (0.053) (*n* = 23)

EPP, end plate potential; MEPP, miniature endplate potential.

Values reported as mean (SEM).

*p<0.05; **p<0.001.

Except for occasional small angular fibres and type I fibre predominance there were no other histological abnormalities. The acetylcholinesterase reaction performed in teased muscle fibre bundles revealed, as in control muscles, only one endplate per muscle fibre; however, the mean endplate area of 169.9 (16.6) μm^2^ (17) in the patient was reduced in comparison with the mean endplate area of 227.03 (11.9) μm^2^ (40) in controls.

**Figure 1:**
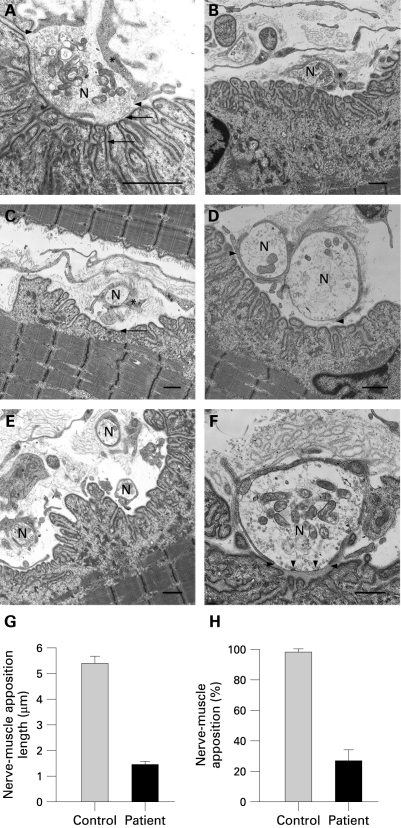
Ultrastructural findings at the neuromuscular junction (NMJ). Control (A) and the patient (B–F). (A) Normal NMJ from a control demonstrating normal nerve terminal size and highly complex postsynaptic membrane folding with well formed secondary synaptic clefts. The arrow heads point to Schwann cell processes, which cap the nerve terminal (N), without extending into the synaptic cleft. The arrows point to the primary synaptic cleft (top) and at a secondary synaptic cleft (bottom). The asterisk in A–C is placed over the Schwann cell. (B) Small nerve terminal partially encased by a Schwann cell process, which intrudes into the synaptic space. (C) Small nerve terminal retracted from the synaptic space and completely engulfed by the Schwann cell. Note also pronounced simplification of the postsynaptic membrane (arrow). (D) Bifurcated nerve terminal with one nerve ending completely engulfed (top arrowhead) and the other ending partially encased (bottom arrowhead) by the Schwann cell. Note also pronounced widening of the primary synaptic cleft and reduction of the density of synaptic vesicles in the nerve terminal. (E) Nerve terminal divided in three small endings, which are encased by the Schwann cell and retracted from the postsynaptic membrane. (F) Pronounced reduction of the area of apposition between the nerve terminal and the postsynaptic membrane, widening of primary synaptic cleft and invasion of the synaptic space by Schwann cell processes (horizontal arrowheads). The vertical arrowheads point to two active zones, which in contrast to the control, are not apposing postsynaptic secondary clefts. (G) and (H) Quantification of the area of apposition between the nerve and muscle. (G) Bar graph representing the average length of apposition between nerve and muscle in 11 controls and 11 patient endplates. (H) Percentages of direct nerve–muscle apposition relative to the total length of the synaptic cleft in 24 controls and 11 patient endplates (mean (SEM)). Calibration marks (A–F) represent 1 μm.

The most consistent structural abnormality of the NMJ shown by electron microscopy were: (1) small axon terminal size and encasement of nerve endings by the Schwann cell; (2) severe widening of the primary synaptic clefts with invasion of the synaptic space by processes of Schwann cells; and (3) moderate simplification of the postsynaptic membranes (fig 1). The small nerve terminals, which often appeared divided into multiple very small segments and retracted from the postsynaptic membrane, were in all cases partially or completely encased by processes of the Schwann cells. As a result of the encasement of the nerve terminal and invasion of the synaptic space by the Schwann cell, the areas of apposition between the nerve terminal and the postsynaptic membrane were extremely small, and even in these areas where presynaptic membranes were directly apposed to postsynaptic membranes the primary synaptic cleft was notably widened. Occasional active zones were seen in the nerve terminal, but they were not consistently apposed to the secondary clefts, as seen in the controls. Some nerve terminals showed a relatively normal number of synaptic vesicles, while others showed few synaptic vesicles. Overall, there was a mild reduction in the density of synaptic vesicles compared to the controls. In the postsynaptic region, there was simplification of the postsynaptic membranes, which was variable and minor compared to the extreme hypoplasia of the axon terminals. There was no other postsynaptic abnormality, with the exception of two sub-sarcolemmal nuclei containing degenerating membranous debris.

The morphometric analysis of the NMJ was performed as previously described^16^ and revealed that relative to the controls, our patient showed diminished average axon terminal area and number of synaptic vesicles per area (table 2). The width of the primary synaptic cleft was notably increased and the area of direct apposition of the nerve terminal to the postsynaptic membranes was extremely small (fig 1). There was also a reduction of the endplate index (ratio of postsynaptic membrane length/presynaptic membrane length), although the average number of secondary folds per μm of primary cleft was not decreased.

**Table 2:** Morphometric data

	Patient	Control
EI*	5.68 (0.66)** (*n* = 24)	11.71 (2.36) (*n* = 12)
Secondary clefts per primary cleft length	1.82 (0.19) (*n* = 24)	1.79 (0.14) (*n* = 12)
Nerve terminal area (μm^2^)	3.73 (0.53)*** (*n* = 24)	7.34 (0.93) (*n* = 12)
Number of synaptic vesicles/μm^2^	9.48 (1.14)** (*n* = 23)	16.77 (2.77) (*n* = 12)
Cleft width (μm)	0.16 (0.030)** (*n* = 11)	0.074 (0.0036) (*n* = 12)

*EI, endplate index (postsynaptic membrane length/presynaptic membrane length).

Values reported as mean (SEM).

**p<0.05; ***p<0.001.

**Figure 2:**
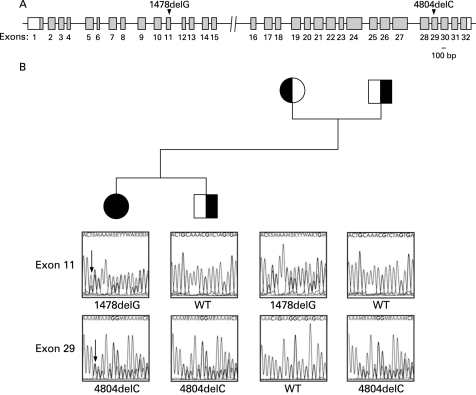
Mutational analysis findings. (A) Schematic view of the 32 coding regions of human *LAMB2* showing the positions of the identified mutations in exons 11 and 29. White regions correspond to untranslated portions of the gene. The 100 bp marker corresponds to exons and introns. (B) Pedigree of the family and the electropherograms displaying the heterozygous frameshifting *1478delG* and *4804delC* mutations in the patient, the heterozygous *1478delG* mutations and a normal sequence or wild type (WT) in the non-affected mother, and the heterozygous *4804delC* mutation and a WT sequence in the non-affected father and brother. The arrows point to the nucleotide deletions.

After obtaining a signed consent approved by the institutional review board of the University of California Davis, we amplified and sequenced genomic DNA from all 32 exons of the human LAMB2 gene and encountered two novel *LAMB2* mutations (fig 2, supplemental table 1). Both mutations are single base pair deletions, resulting in frameshifts. *1478delG* occurs in exon 11, while *4804delC* occurs in exon 29. *1478delG* results in an early stop codon at amino acid 496, and *4804delC* creates a termination codon at amino acid 1653. Mutational analysis in the patient’s family revealed that the *1478delG* mutation derives from the patient’s unaffected mother. In contrast, the *4804delC* mutation is derived from the patient’s unaffected father and is also carried by the patient’s unaffected brother. Mutational analysis in *CHRNA1, CHRNB, CHRND*, CHRNE, *RAPSN, MUSK, DOK7* and *COLQ* was performed as previously described^16^ and showed no abnormalities.

**Figure 3:**
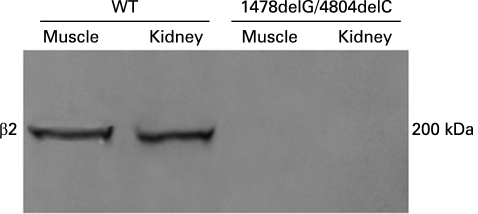
Western blot analysis. Results of a Western blot using a rabbit polyclonal antibody directed against an epitope corresponding to amino acids 1549–1798 of human laminin β2 (Santa Cruz Biotechnology, Santa Cruz, California, USA) and frozen tissue from renal and muscle biopsies of the patient. There is no laminin β2 expression in muscle and renal tissues from the patient.

Because both genetic defects encountered in *LAMB2* are deletions leading to frame shifts, we predicted that the protein would be truncated and fail to assemble with the rest of the subunits. Indeed, both Western blot, performed as previously described,^17^ in kidney and muscle tissues, and immunohistochemistry in kidney tissue showed no expression of laminin β2 (fig 3, supplemental fig 1).

## DISCUSSION

Since each of the non-affected parents and the brother of the patient carry only one of the mutations, the pattern of inheritance of CMS associated with *LAMB2* mutations is autosomal recessive, as is the case with the majority of other forms of CMS.^1^ ^2^

While humans with pathogenic *LAMB2* mutations have been found to show the classical renal and ocular dysfunction of Pierson syndrome, a missing piece of the puzzle has been the absence of a clear demonstration in humans of the NMJ involvement predicted by the animal model. This is likely due to the short life span of patients with truncating *LAMB2* mutations, as renal failure usually causes death within the first year of life. Interestingly, longer survival rates in patients with Pierson syndrome have recently been described; however, in the majority of these cases, missense mutations were involved.^18^ ^19^ Since missense mutations would predict some level of expression of synaptic laminins, it is possible that a minimal level of expression of laminin may be sufficient to rescue the neuromuscular phenotype.

The exceptional feature of our patient is that she has had an unusually long life as a result of a successful renal replacement therapy using a highly compatible kidney donated by her father (she is still alive and well at the age of 20 years). This has allowed the clinical manifestations of the failure of neuromuscular transmission to emerge.

In terms of the pathogenesis of this CMS, the structural changes and the functional abnormalities of the NMJ encountered in our patient are strikingly similar to those reported in *Lamb2^−/−^* mice. Thus, the extensive analysis performed in *Lamb2^−/−^* and the conclusions drawn from this animal model provide an invaluable key to understanding the pathogenesis of the human neuromuscular disease reported here. First, as in *Lamb2^−/−^* mice, our patient showed immature hypoplastic nerve terminals, which in the animal model have been attributed to the fact that laminin β2 acts as a potent “stop signal” for motor neurite growth and promotes presynaptic differentiation in vitro.^15^ ^20^ ^21^ It follows that in the absence of laminin β2, nerve terminals fail to mature, remain hypoplastic and become encased by cytoplasmic processes of the Schwann cell. The abnormalities of the active zones that we encountered in our patient have also been seen in the animal model^22^ and have been attributed in part to a lack of interaction between the β2-chain containing laminin 9 (α4/β2/γ1) with the pore-forming (Ca_v_) subunit of the voltage gated calcium channel.^23^ ^24^ This decrease of active release sites, along with the reduction of nerve terminal size and the dramatic decline of the area of apposition between the nerve terminal and the postsynaptic membrane, accounts for the distinctive depression of the EPP quantal content and MEPP frequencies that characterise both the human disease and the animal model.^21^ Second, the invasion of the synaptic space by Schwann cell extensions found in our patient and in *Lamb2^−/−^* mice has been interpreted in the animal model as resulting from the loss of the inhibitory effect that the β2 chain containing laminin-11 exerts on Schwann cell propagation.^20^ Third, in our case, as in *Lamb2^−/−^*, there was a simplification of the postsynaptic folding pattern. This simplification of postsynaptic folds may result from the lack of an interaction between β2 chain containing laminins and cell surface receptors for laminins such as integrins and dystroglycan that participate in the development and maintenance of the synaptic scaffold.^11^ ^13^

**Web resources**

The URLs for data presented herein are:

- National Center for Biotechnology Information (NCBI): http://www.ncbi.nlm.nih.gov/
- Online Mendelian Inheritance in Man (OMIM): http://www.ncbi.nlm.nih.gov/Omim/
- Basic Local Alignment Search Tool (BLAST): http://blast.ncbi.nlm.nih.gov/Blast.cgi
- Entrez Single Nucleotide Polymorphism Database: http://www.ncbi.nlm.nih.gov/sites/entrez

Using tissue specific laminin β2 transgenes, it was recently demonstrated that salvaging the glomerular defects with a podocyte specific β2 transgene (NEPH- β2) does not significantly improve the phenotype of the *Lamb2^−/−^* mutant, while rescuing the structure of the NMJ with a muscle specific β2 transgene (MCK-B2) extends life by 50%.^25^ Clearly in the animal model, defects of the NMJ are more devastating than kidney abnormalities.

Since we see no obvious central nervous system defects in humans, and since the ocular abnormalities are not lethal, the NMJ disease may become the most serious complication in patients with biallelic truncation mutations in *LAMB2* and long survival due to successful renal replacement therapy. From a therapeutic perspective, drugs that increase the release of neurotransmitter from the nerve terminal, such as 3,4-diaminopyridine, may be beneficial, but should be given with caution to patients who, like ours, have profound distortion of the function and architecture of the NMJ.
